# StaticPigDet: Accuracy Improvement of Static Camera-Based Pig Monitoring Using Background and Facility Information

**DOI:** 10.3390/s22218315

**Published:** 2022-10-29

**Authors:** Seungwook Son, Hanse Ahn, Hwapyeong Baek, Seunghyun Yu, Yooil Suh, Sungju Lee, Yongwha Chung, Daihee Park

**Affiliations:** 1 Department of Computer Convergence Software, Korea University, Sejong 30019, Korea; 2 Info Valley Korea Co., Ltd., Anyang 14067, Korea; 3 Department of Software, Sangmyung University, Cheonan 31066, Korea

**Keywords:** pig detection, image processing, deep learning, video monitoring, static camera, background, facility, occlusion

## Abstract

The automatic detection of individual pigs can improve the overall management of pig farms. The accuracy of single-image object detection has significantly improved over the years with advancements in deep learning techniques. However, differences in pig sizes and complex structures within pig pen of a commercial pig farm, such as feeding facilities, present challenges to the detection accuracy for pig monitoring. To implement such detection in practice, the differences should be analyzed by video recorded from a static camera. To accurately detect individual pigs that may be different in size or occluded by complex structures, we present a deep-learning-based object detection method utilizing generated background and facility information from image sequences (i.e., video) recorded from a static camera, which contain relevant information. As all images are preprocessed to reduce differences in pig sizes. We then used the extracted background and facility information to create different combinations of gray images. Finally, these images are combined into different combinations of three-channel composite images, which are used as training datasets to improve detection accuracy. Using the proposed method as a component of image processing improved overall accuracy from 84% to 94%. From the study, an accurate facility and background image was able to be generated after updating for a long time that helped detection accuracy. For the further studies, improving detection accuracy on overlapping pigs can also be considered.

## 1. Introduction

Over the years, the demand for pigs has increased worldwide. According to the OECD, the global pork consumption rate, in tons, has increased from approximately 63,000 kilotons in 1990 to 108,000 kilotons in 2021 [1]. As demand rises, the number of pigs within each farm increases, accordingly, thereby increasing the difficulty of pig management. Thus, managing each pig individually to their health and welfare needs is not an easy task. To reduce management workload, many studies have reported the use of surveillance techniques to address health and welfare problems [2,3,4,5,6,7,8,9,10,11,12,13,14,15,16,17,18,19,20,21,22,23,24,25,26,27,28,29,30,31,32,33,34,35,36]. Therefore, the use of object detection [37] to detect pigs by means of a surveillance camera can reduce the management workload within a pig pen.

**Figure 1:**
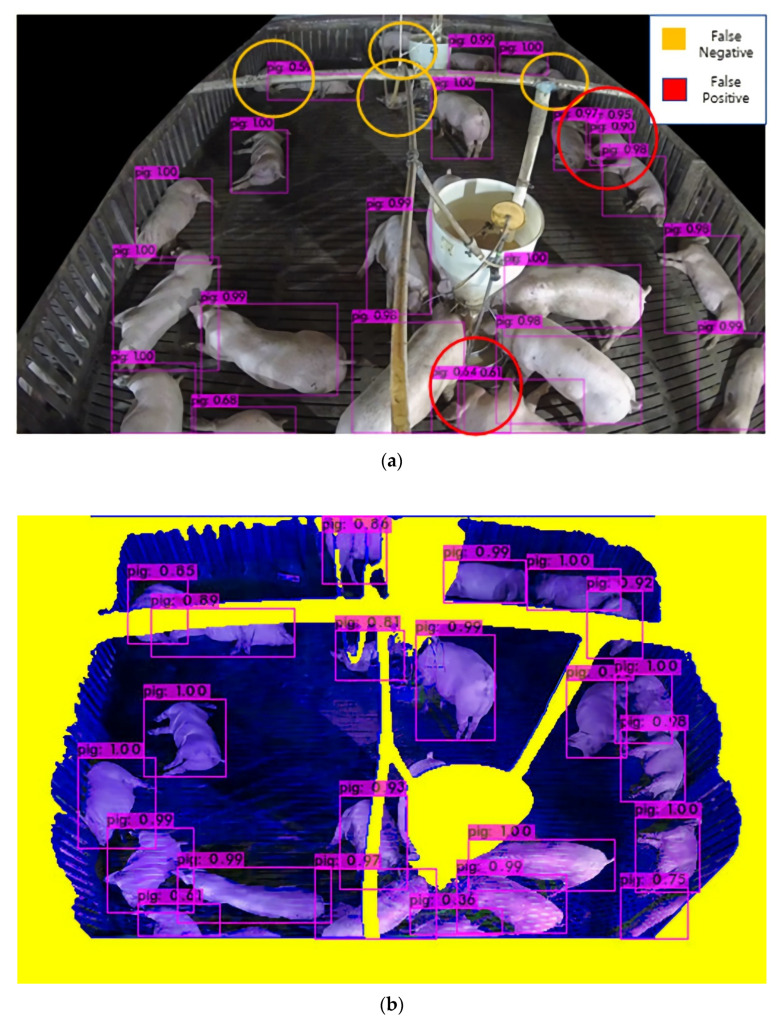
The pig detection results for a commercial pig pen with a tilted-view static camera.

Single-image object detection technology can effectively enable pig detection, as it exhibits significant improvement over the years of technological advances. Approaches such as YOLO [38,39,40,41,42], which satisfies real-time detection speed on an embedded board, improve detection accuracy in certain cases wherein target objects are non-occluded and sufficiently large. However, the object detection technology locates the appearance of each object within an image [38]. Consequently, whenever a pig object is occluded by a complex facility (e.g., a feeder), it cannot be sufficiently identified by existing object detectors, thus reducing the overall detection accuracy [27,34,35,36]. As pigs are regularly within the proximity of feeder facilities for nourishment, they are frequently occluded in pig pen images [11,28]. Other large objects, such as ceiling pipes that connect feeder facilities, may also occlude pigs. Object detection challenges primarily occur owing to differences in pig size and facility occlusion (Figure 1). For explainability purposes, the object detector used throughout this study is referred to as tinyYOLOv4 [41] although the proposed method can be applied to any deep learning-based object detector.

In an actual Hadong farm, since a top-view camera that covers an entire pig pen is difficult to install, a tilted-view camera is installed. However, object detection difficulty arises from differences of pig sizes by the distance from the pig and the camera or occluded pigs by feeder or facility. As shown as Figure 1a, if tinyYOLOv4 is used to process pig object detection, pigs that are far in distance from the camera, object detection is difficult as the pig object size is small. Additionally, different errors occur for cases like pigs that are close to the camera as even they create two detection boxes for a pig or facility occluded pigs create detection errors. As shown as Figure 1b, the proposed method can solve the error cases due to the difficulties on pig size differences and object occlusion by transforming perspective and identifying the location of the facility.

Because the cameras installed in pig pen are static, stationary facilities and objects (e.g., walls, feeders, and pipes) remain constant throughout the footage. Therefore, the stationary facility object like walls, feeder, or pipes all stay constant within the long period of time that the camera films. However, pig objects themselves continuously move and change position. Although the improvement from updating the background and facility information for each frame may be miniscule, accuracy may increase substantially as these changes accumulate. Therefore, the continuous fine-tuning of the background and facility information can improve object detection performance [25,29]. Furthermore, as the duration of footage increases, the accuracy of extracted information should also increase.

If the deep learning model can specify locations of occluding objects within a surveillance image, as well as learn the corresponding information, detecting pigs behind those objects is possible [25,29]. We therefore present methods for deep learning-based object detection utilizing extracted background and facility information from images of a pig pen environment that contains various complex structures. The method includes a process that resizes different pig objects through the warp perspective method, receives the results of object detection, uses those results in image processing, and reuses the results of image processing to supplement object detection, thereby improving accuracy. Specifically, the system continuously receives box-level object detection results using a deep-learning-based detector from video data generated via static camera, updates the background extraction parameters, and acquires continuously improved pixel-based background images.

The extracted information is used to create different combinations of gray images. Subsequently, the gray images are combined into different combinations of three-channel composite images, which are used as training datasets to improve detection accuracy. The original image’s facility texture is one-channel, and usually colored similarly to flooring. However, by adjusting the color compositions of the background, facility, and foreground, deep learning can successfully differentiate between the corresponding features, thus increasing detection accuracy. The input image (Figure 1b) of the object detector is altered to mitigate the error caused by differences in pig size and occluding objects.

Therefore, main objective of the study is to improve the detection accuracy of pig objects occluded behind facility and small objects that are located far from the source camera by revising the pig object sizes to be similar in size and generating background and facility image created from a video recorded on a static camera of the environment for a long period of time

## 2. Related Works

This study aims to solve the accuracy reduction problem that occurs from object occlusion. Although many studies have been conducted to improve pig monitoring technology within a pig pen, many environmental variables are involved. Early studies focused on improving pig monitoring using image processing methods. The detection of pigs within images at the pixel level [4,5] was considered. Moreover, 24 h surveillance of pig movements has been attempted via video sensors [6,7] and a similar approach was employed to estimate the locomotion of pigs within a pen [8]. However, factors such as differences in lighting conditions [9] may interfere with foreground detection. The adaptive thresholding of an image for foreground detection has also been introduced [10]. Aggressive behavior [11,12] or any movement with an angular histogram [13], was examined. The detection of multiple pigs standing still within a pig pen can be achieved in different ways [14,15].

As deep learning methodology has improved over the years, the use of this technology in pig monitoring has increased. The detection of pigs under surveillance video [16] with suboptimal conditions [17] was studied to improve accuracy. Attempts have been made to detect posture [18] and count each pig within a pen [20] using deep learning. This technology allows for improved management of health issues in pigs [22,23,24,26]. More sophisticated methods have been introduced to address pig monitoring issues [27,31,32]. For instance, detection of pig posture was studied on a more specific level [28,33]. Testing the pig object detection under lighter hardware is also considered for overall process speed [34]. Reducing image noise can increase overall image quality, thus improving the detection accuracy for pig monitoring [35,36]. There are more pig farm images on different pig pen environments like a camera is installed with top-view [19] or tilted-view [21,30], but facility has not presented. There are also researches that detect pigs occluded by facility with tilted-view as well [25,29], but background and facility information has not been utilized.

While many studies improve pig monitoring within a pig pen with their individual methods, our method uses a static camera that fine tunes different images that can be used to identify different aspects within a pig pen (i.e., facility and background. Most other studies use single “independent” image to improve pig monitoring, but our method utilize the characteristics of “continuous and consecutive” images (i.e., video) that have static background and facility. Recent studies that dealt with pig detection methods chronologically is as shown as Table 1.

## 3. Proposed Method

Occlusion of pigs behind objects or facilities leads to detection errors. To solve the issue, this study proposes a method that solve accuracy reduction caused by facilities within a pig pen using image processing methods. To obtain the detection boxes, the object detector is applied to the input image from a continuous video feed recorded by a static camera. The pixel-level background and facility images are then continuously improved using detection boxes. Finally, composite images are created to train an object detector.

A long surveillance video can be deployed on a pig pen to achieve continuous fine-tuning, wherein each video frame updates the background and facility images by a small amount. Each small updates are built up to be more accurate background and facility images by changing the background and facility images by one pixel value for all the pixels within an image with the proposed method.

We used the background and facility images to build composite images trained for tinyYOLOv4. Composite images may be categorized as one-channel or three-channel. A one-channel composite image is obtained by extracting foreground, background, and facility information by manipulating pixels, thus granting the benefit of identifying its location and differentiating its information. A three-channel composite image is a concatenation of three one-channel composite images, allowing for more diversity in the textures of target objects.

**Figure 2:**
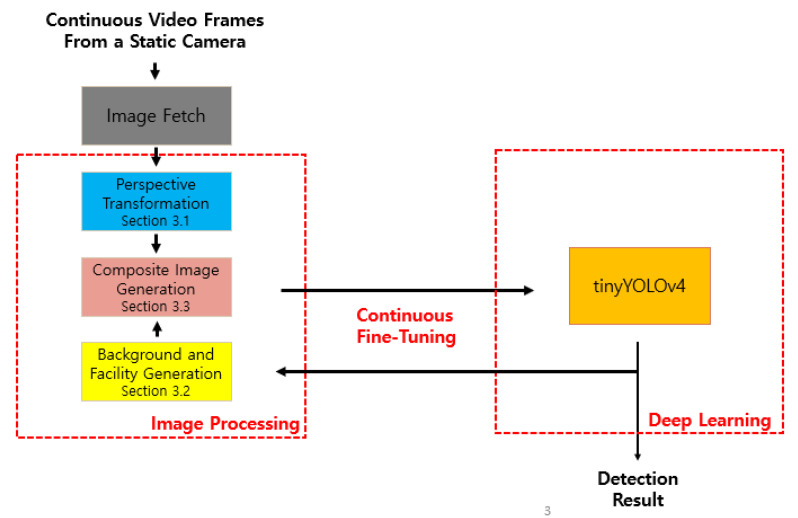
Overview of proposed method StaticPigDet.

The proposed composite image framework enables tinyYOLOv4 to efficiently learn features of pigs occluded by facilities, thus increasing the detection accuracy of all pigs regardless of occlusion. Figure 2 illustrates the overall structure of the proposed method; wherein composite images are generated to minimize false-negative and false-positive errors. The figure shows that this method continuously improves the accuracy of image processing and deep learning.

### 3.1. Perspective Transformation

**Figure 3:**
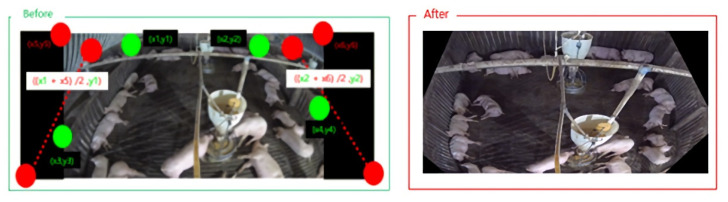
Image of original image and perspective transformation applied image. Left image represents input image filmed from surveillance camera with line to apply perspective transformation on. Right image represents aftermath of perspective transformation method.

**Figure 4:**
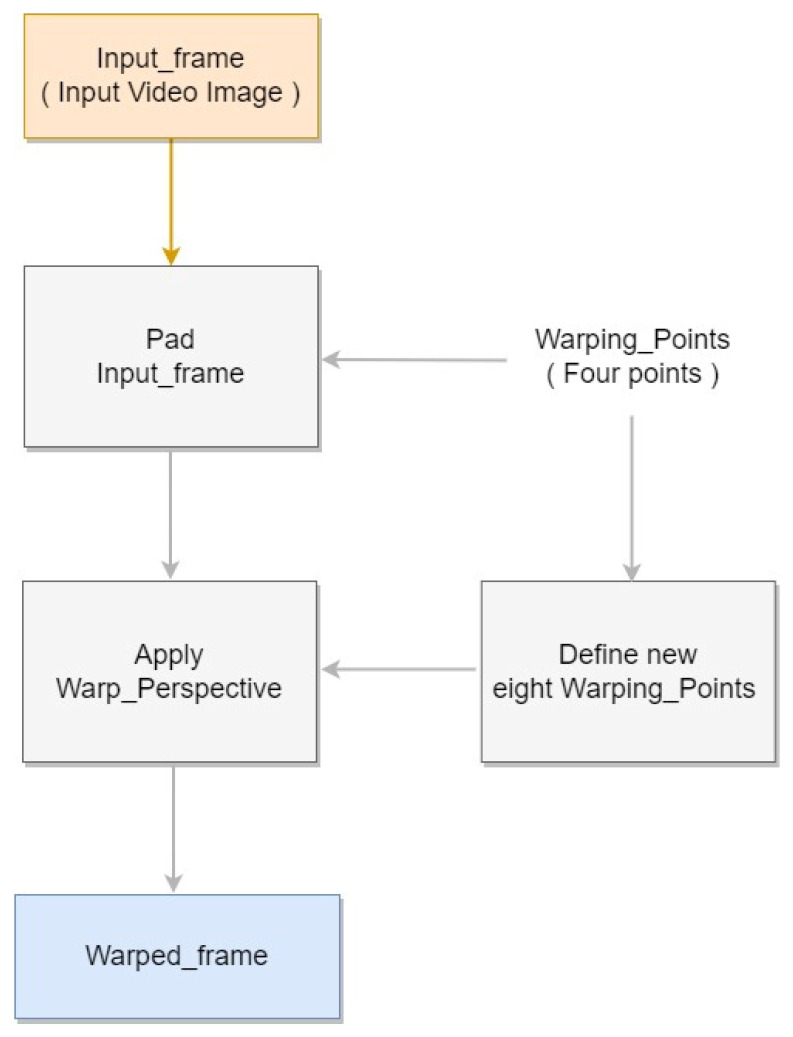
Perspective Transformation block diagram.

One obstacle that hinders accurate object detection is the differences in size of target objects owing to distance from the camera. To mitigate this issue, we incorporated an automatic perspective transformation. The warp perspective transformation method [43], an image processing method, deforms the input pixel grid to fit the output pixel grid by changing the sizes of pixels within. With this process, the object detection difficulty due to differences in pig sizes was alleviated. To establish the transformation points, we applied our automatic perspective transform method. First, we calculated the slopes of the warping points on either side. We then padded the intersection points between the slope and the top and bottom edges, where the original image’s resolution is exceeded. These locations were used to select new warping points. Finally, perspective transformation was ap-plied to the eight new warping points to generate a new transformed image. The result is shown in Figure 3, and Figure 4 shows the block diagram corresponding to the Perspective Transformation.

### 3.2. Background and Facility Generation

In this study, locating the background and facility was an essential step for extracting the corresponding textures. However, identifying an object from a single image is tricky task. Generally, pig objects within a pig pen exhibit passive, if any, movement. Given this characteristic, the background and facility images can be improved via gradual changes. After a certain frame, a sufficient level of background and facility images can be generated.

**Figure 5:**
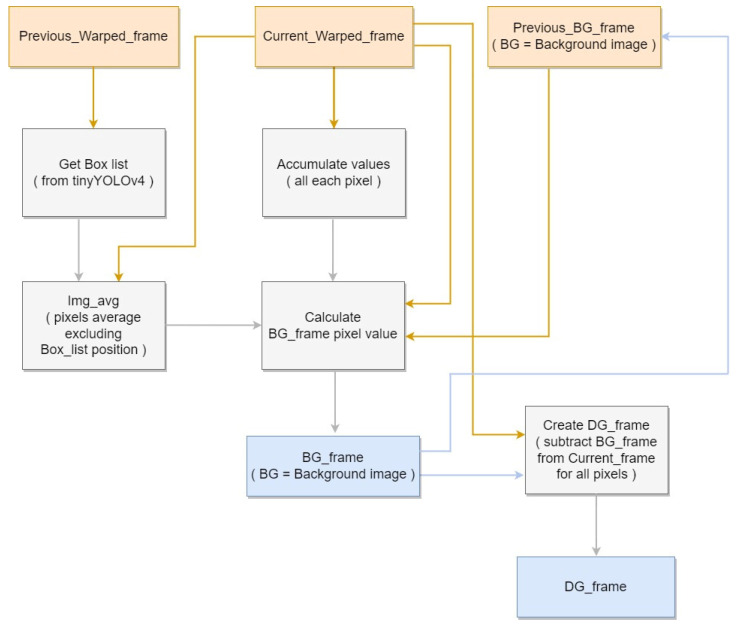
Background Generation block diagram.

Subsequently, pixels that continuously appear within detected boxes are classified as not affecting the background and are substituted with the current frame’s average pixel. However, as the actual background does not remain fixed, the background image needs to be gradually updated according to each subsequent frame. To settle the issue, each pixel on current frame, excluding the detection box region, is compared with previous frame’s each pixel. If current frame’s pixel is higher than previous frame’s pixel on the same location, then background’s pixel is raised by one, otherwise if less, lowered by one. With long period of frames, background image can be generated by using tinyYOLOv4 detection result using video data that contains pig objects. Finally, we generate the difference image by calculating difference between pixel on current frame image and current frame’s background image. The image is necessary to use on facility image generation module to identify the location of foreground on pig object. Figure 5 shows the block diagram corresponding to the Background Generation.

As explained previously, false positives may occur for objects occluded behind facilities. Accordingly, this paper proposes an image generation method to identify facility locations using the proposed background generation approach. Within each image, a facility is defined as a region where pigs are not potentially located owing to occlusion. As visually identifying the presence of occluded objects is difficult, the region information of the difference image is employed.

**Figure 6:**
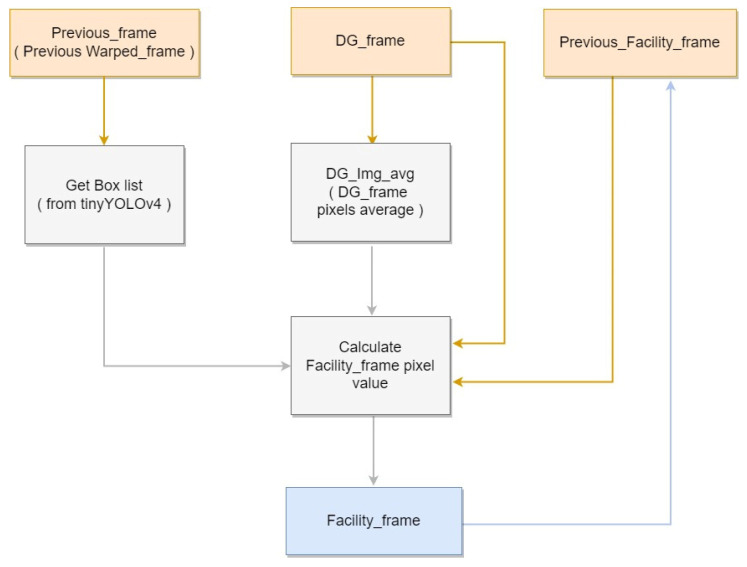
Facility Generation block diagram.

First, the initial pixel value of the entire facility image is set to the maximum value of 255. Subsequently, the average pixels of the entire difference image, as well as those of its detected box regions, are calculated. Generally, average pixel values within pig object regions are higher than that of the background region. Therefore, if a box region’s average pixel is lower than that of the overall image, the detected box may be a false positive, and should be exempted from the update. If a pixel value within the box is higher than the box’s average, the pixel value in the facility image is reduced by 1, as it is considered a pig region. Furthermore, all foreground pixels in the pig region are set to 255 and 0 if otherwise. As the generated background image may contain noise, and cannot be perfectly identical to the current background, calibration is performed on a certain interval of frames. If insufficient pixel changes occur, the result can be considered temporary noise. Therefore, any pixel values higher than 245 are reset to 255, along with their immediate neighboring regions. For the experiment, the interval was set to 10,000 frames. Figure 6 shows the block diagram corresponding to the Facility Generation.

### 3.3. NPPS and Composite Image Generation

**Figure 7:**
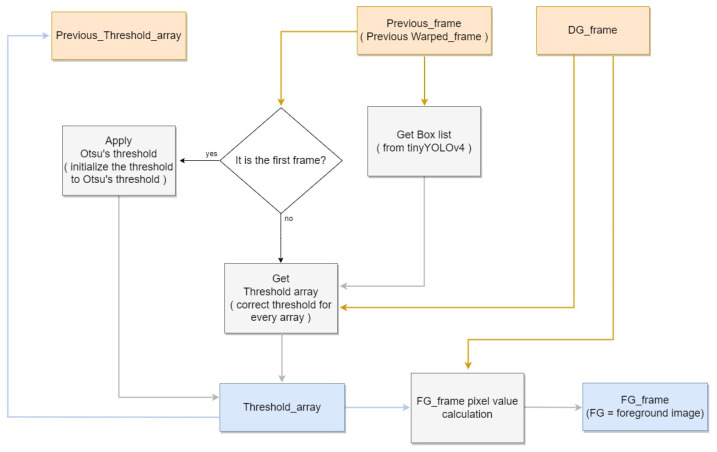
Non-Pig Pixel Suppression block diagram.

After the difference image is generated, foreground images can also be generated on the same frame. These images are used to locate pig objects with respect to pixels. Consequently, not only are pig objects identified but non-pig regions can also be suppressed using the thresholding technique. This technique sets a certain global threshold for pixel values to differentiate between background and foreground pixels. We propose the non-pig pixel suppression (NPPS) method, which sets individual thresholds for each pixel. This enables adaptive thresholding in accordance with different environmental variables, such as lighting. In the first frame of the image, all thresholding is initialized using Otsu’s algorithm. Detection boxes are then used to determine whether each threshold should be incremented or decremented. If a pixel within the detection box has a higher value than its corresponding difference image pixel, the threshold decreases by 1. Conversely, if a pixel outside the detection box has a lower value than its corresponding difference image pixel, the threshold increases by 1. If the threshold is outside the appropriate range, pixels may be incorrectly identified. Therefore, minimum, “min_thresh”, and maximum, “max_thresh”, threshold values were set as limits to the threshold range. Figure 7 shows the block diagram corresponding to the NPPS.

Using the generated background, foreground, and facility images, we implemented composite image generation to eliminate false positives resulting from occluded objects. Subsequently, the contrast-limited adaptive histogram equalization (CLAHE) method [44] was employed to maximize the pixel differences between the background and pig objects. To apply CLAHE, we set ClipLimit (a threshold value for the histogram smoothing process) to 0.6, and TilesGridSize (which determines the block sizes to be divided) to (2, 2), in accordance with [27]. Then, a composite image was generated for training.

All generated images were padded by 32 pixels to replicate occluding walls at the bottom of the image. Although CLAHE was applied to Image A, pixels corresponding to the facility and padding region were set to 255. The CLAHE-applied image replicates the effect of illumination as the average foreground pixel value increases. Thus, the facility is set to 255 for convenience. Image B exhibits a difference in time, and the pixels corresponding to the facility and padding region were set to 0. The difference image is used to suppress the background, thus emphasizing the pig object. Because Image C corresponds to the foreground, all foreground pixels were reset to their original values, whereas those in the padding region were set to 0. Thus, all background and facility effects were removed to isolate the original pig object. Image D is an inverted foreground image, where foreground pixels were set to 0 and all other pixels were reset to their original values. Thus, the shape and edges of the foreground were emphasized while learning the flooring texture.

**Figure 8:**
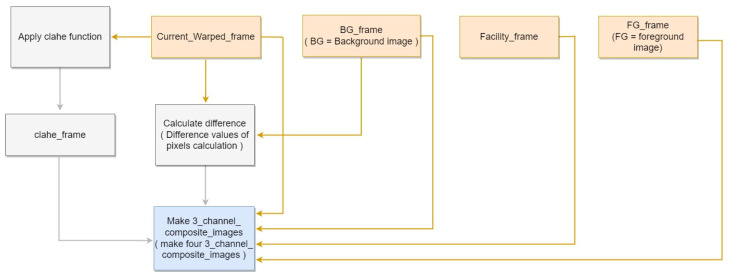
Composite Image Generation block diagram.

The 3-channel composite image is made from selecting three 1-channel composite images from the four proposed images and concatenating them channel-wise. This method allows one image to contain the benefit of three 1-channel images during training. The proposed four 1-channel composite images and the 3-channel composite image generated from concatenating three 1-channel combination. Figure 8 shows the block diagram corresponding to the Composite Image Generation.

## 4. Experimental Results

**Figure 9:**
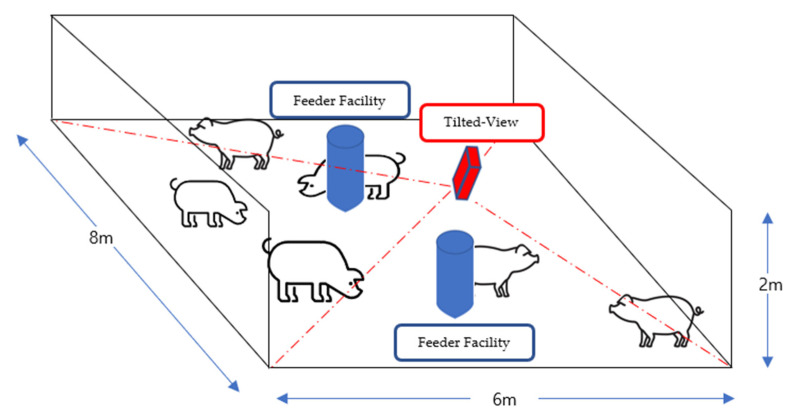
Experimental setup with a tilted-view surveillance camera (shown as red color) to cover a pig pen with feeder facility (shown as blue color).

This experiment was conducted on the Barun pig pen, located in Hadong-gun, Gyeongsangnam-do, Korea and all the video data were obtained from the scenarios scheduled by the commercial pig pen, not from any artificial scenario for this study. Seventy pigs were monitored by a camera with a range that encompasses half the pig pen, split diagonally (Figure 9). All data were collected using a Hanwha QNO-6012R [45] surveillance camera, at a height of 2.1 m on a pole in the center of the pig pen, pointing approximately 45° obliquely. Video data with 1920 × 1080 resolution were acquired at a speed of 30 FPS (Frames per Second), and the Warp Perspective image processing technique [43] was applied to regularize the size of pig objects. The training dataset contained 1600 composite images, whereas the testing dataset contained 200 composite images. To avoid overfitting, the train and test dataset was divided randomly. The ratio of the dataset was 8:1 with train and test, respectively (1600:200 in terms of images). In addition, multiple composite images were used with foreground/background/facility information and trained them as explained in Section 3.3. To remediate the time cost associated with processing, all images were resized to a 512 × 288 resolution, meeting the real-time requirement of 30 FPS. The deep learning model was trained on a PC with an AMD Ryzen 5950x 16-core processor, GeForce RTX 3090 (4352CUDA cores, 11GB VRAM) GPU, and 32 GB of RAM, in Ubuntu 18.04-LTS OS. To train the model, the number of iterations was set to 6000, and learning rate was set to 0.00261. The model was tested on a Jetson TX-2 [46] dual-core Denver 2 64-bit CPU, quad-core ARM A57 complex, NVIDIA Pascal™ architecture with 256 NVIDIA CUDA cores, and 8 GB 128-bit LPDDR4 to test its performance on an embedded board.

**Figure 10:**
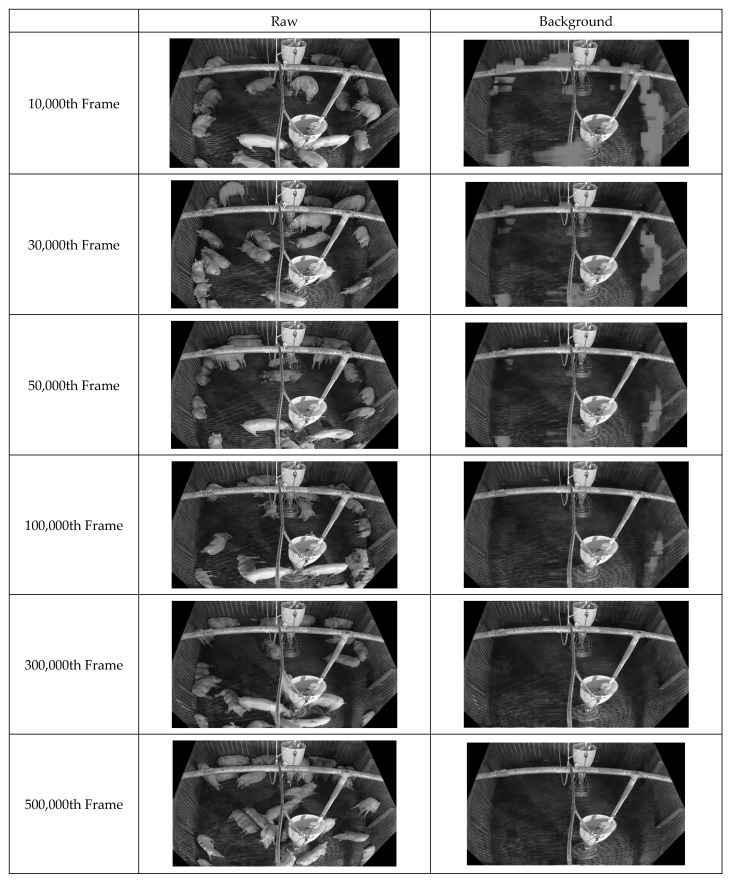
Illustration of Background Image Generation of 500,000 frames with showing each consecutive 20,000 frames, 200,000 frames.

**Figure 11:**
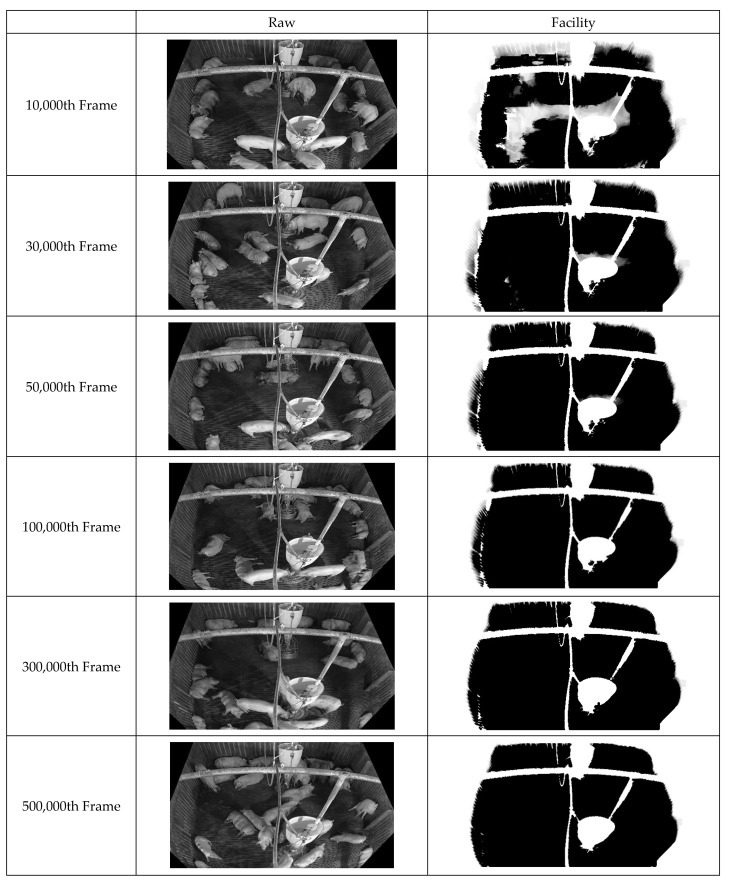
Illustration of Facility Image Generation of 500,000 frames with showing each consecutive 20,000 frames, 200,000 frames.

**Figure 12:**
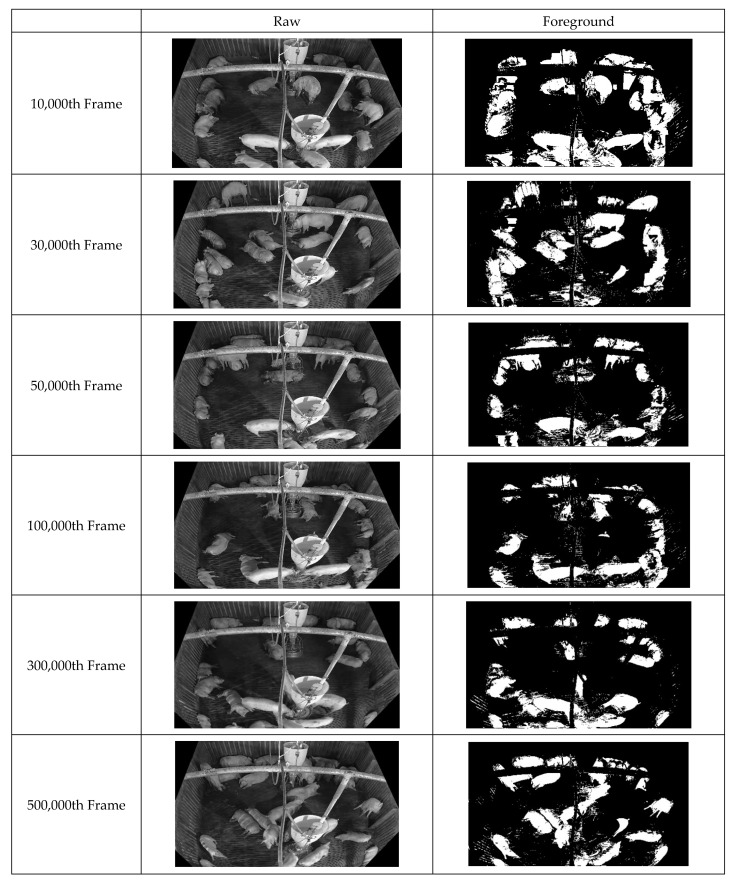
Illustration of NPPS of 500,000 frames with showing each consecutive 20,000 frames, 200,000 frames.

First, improved pixel-based background images were acquired by continuously inputting the box-unit object detection results through applying tinyYOLOv4 [41] to the video data. Figure 10 illustrates the background updating over time. Evidently, the background image gradually becomes clearer as noise is removed. Figure 11 displays the facility information collected from the pig pen. The algorithm for facility generation (see Figure 6) corrects the gaps and unclear contours caused by noise, thus improving the clarity of the facility image. Finally, a foreground image corresponding to the pig object is generated using the input and background images. Figure 12 indicates that this image also improves over time as the background image becomes more accurate. This pattern illustrates how the background image accuracy affects the foreground image accuracy.

**Figure 13:**
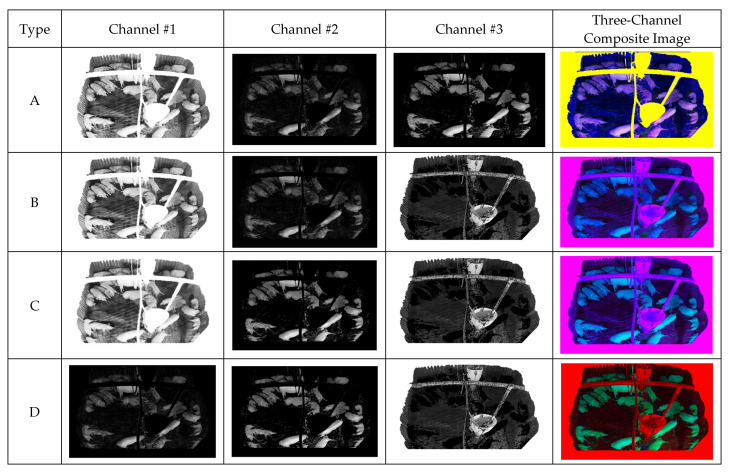
Combinations of 3-channel composite images.

Figure 13 displays the results of four one-channel images generated from the input, background, foreground, facility, and CLAHE images. As 3-channel composite image is generated from concatenating three 1-channel images, the generated three-channel composite image exhibits different colors (Figure 13). Each one-channel image is shown via gray channel, whereas each three-channel image is shown via color channels.

Table 2 and Table 3 present an accuracy comparison between the baseline (tinyYOLOv4 [41] and tinyYOLOv7 [42]) and proposed StaticPigDet. AP0.5 (average precision with 0.5 IoU) is a performance index used to measure object detection accuracy in benchmarks such as PASCAL VOC. Specifically, it indicates the average precision based on an intersection over union (IoU) of 0.5. Precision is calculated using the TP (true positive) and FP (false positive) cases, whereas recall is calculated using the TP and FN (false negative) cases. Subsequently, the average precision based on IoU is calculated using the inverse properties of precision and recall.

Within the training dataset, “Color Image” denotes the original color images obtained by camera, whereas “Composite Image” comprises the images generated by the composite method. The model is tested on each three-channel composite image types and each 3-channel composite image dataset contain 200 images, which is each named “Composite Image A”, “Composite Image B”, “Composite Image C”, “Composite Image D”. The “Composite Image A + B + C + D” includes a total of 1600 images, with four images for each of “Composite Image” subsets forementioned and four images for each of four one-channel images generated from the input as mentioned in Figure 13. The model was tested on the reconstructed three-channel composite images. As a result, the proposed method improved accuracy by 5–10% on the overall test dataset compared to the baseline models. Likewise, overall TP, FP, FN, precision, and recall also improved.

**Figure 14:**
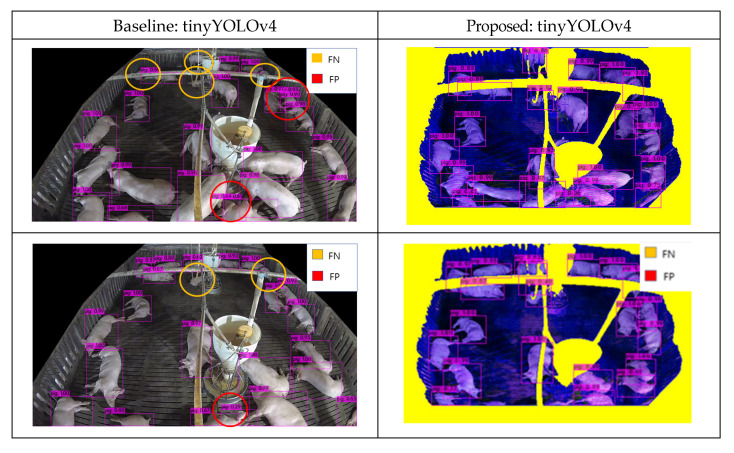

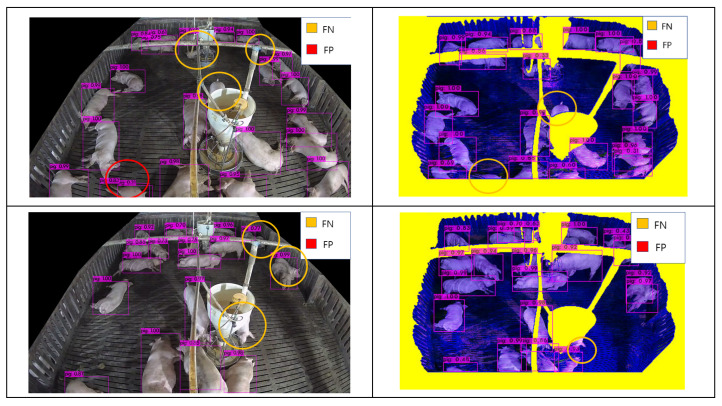
Detection results for Hadong with object detectors [41] (Baseline vs. Proposed) that shows solved cases and unsolved cases.

Many images collected from surveillance camera create different error cases. These error cases include pig object occlusion behind feeder facility and occlusion behind ceiling pipes. Many pig objects were not detected, resulting in FN results. By applying the composite image methodology, most, but not all, of these errors were resolved. Figure 14 presents the detection results for Composite Image A, wherein each error case was designated from the results. Although cases of body-separated occlusion wherein a pig object was split by an occluding feature, were handled accordingly, cases where part of the pig was cut off entirely, or pigs occluded each other, remained causes of error.

For further analysis, we trained 200 composite images of each type to examine their individual effects on training. Overall detection accuracy was higher than that of the baseline color images but lower than that of the composite image dataset (See Table 4 and Table 5).

**Figure 15:**
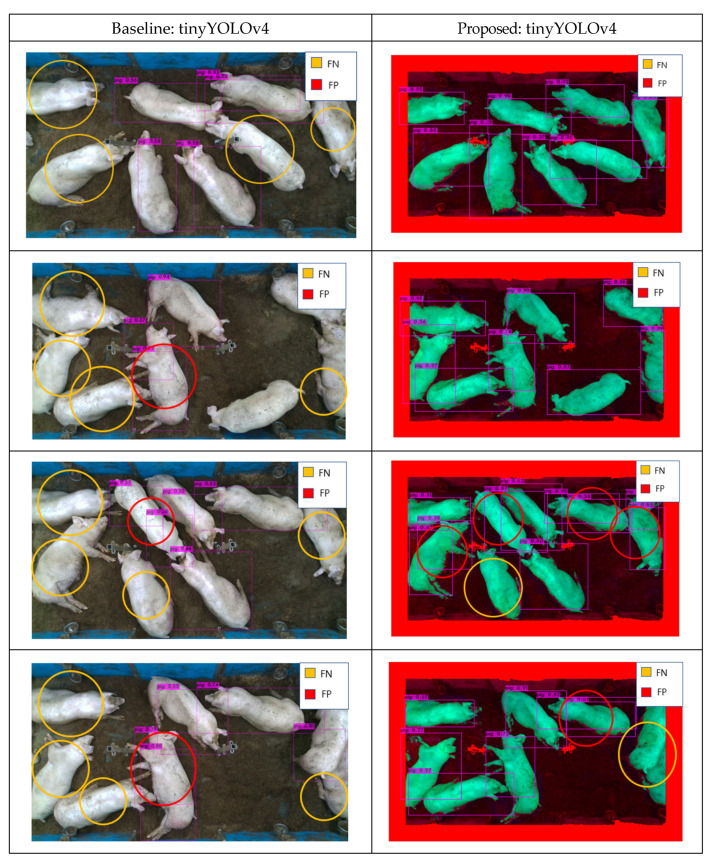
Detection results for Chungbuk with object detectors [41] (Baseline vs. Proposed) that shows solved cases and unsolved cases.

As pig pen environments exhibit significant differences, an effective deep learning model should achieve sufficient accuracy for different pig pen. To evaluate our proposed method’s robustness, we tested all models on pig pen images taken from the Chungbuk National University. The pig pen comprised a 4.9 m × 2.0 m × 3.2 m tall pigsty. To obtain the images, we installed an Intel RealSense camera (D435 model, Intel, Santa Clara, CA, USA) [47] on the ceiling. Table 6 and Table 7 present a detection accuracy comparison on training from the Hadong dataset and testing on the Chungbuk dataset. Although latter environment did not feature significantly occluding feeding facilities, deep learning models trained from the Hadong dataset still showed decreased object detection accuracy for the Chungbuk dataset. Compared to the baseline model, however, the proposed method could increase the detection accuracy up to 15% (from 75.86% to 90.25% with tinyYOLOv4, from 63.15% to 77.76% with tinyYOLOv7), thus showing the proposed method’s robustness on other pig pen environment. Each detection cases for Chungbuk test dataset shows improvement, but not all cases are solved as shown as Figure 15.

FPS (Frames Per Second) is widely used as an execution speed for video applications, and higher speed in FPS implies a faster processing speed. In other words, if the execution time of object detection is 30 FPS or more, it is considered as real-time. Finally, the result of multiplying the accuracy and execution speed can be used as an integrated performance index, wherein a higher value indicates a higher integrated performance.

**Figure 16:**
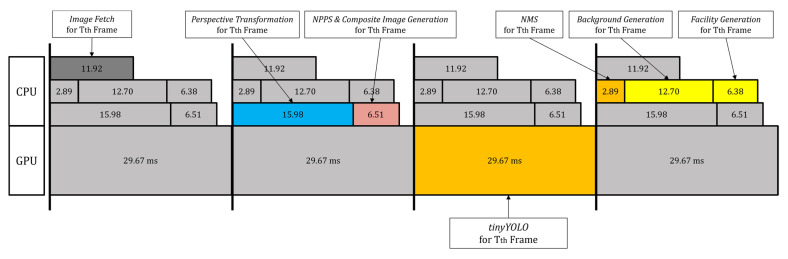
Illustration of the code structure for a pipelined execution, along with execution times for each step, with tinyYOLO on a TX-2 [46].

For each video frame, we need to execute Image Fetch (on CPU), Perspective Transform and NPPS & Composite Image Generation in the proposed method (on CPU), tinyYOLO (on GPU), NMS (i.e., Non-Maximum Suppression to delete similar boxes [38,39,40,41,42] (on CPU), and Background Generation and Facility Generation in the proposed method (on CPU), sequentially. For real-time processing, we use multi-core programming (on a multi-core CPU) and pipeline techniques to overlap multiple frames processing. With the incorporation of multi-core programming and pipeline techniques as shown in Figure 16, the CPU and GPU computation can be overlapped over multiple frames thus raising throughput on continuous video frames.

Table 8 compares the integrated performance of tinyYOLOv4 and tinyYOLOv7. Compared to the baseline model, the proposed method increases accuracy under real-time requirements, which yields an improved performance on a TX-2 board [46]. This indicates that the proposed method can be implemented on an embedded board to achieve a real-time requirement of 30 FPS while improving accuracy.

## 5. Prospects for Further Research

While the study mainly solves issue of pigs occluded by facility within a pig farm, there are many issues still left to solve. One of the issues on decreasing detection accuracy is occlusion caused by multiple pigs overlapping each other. Figure 15 shows many FP created from pigs overlapping one another and sometimes creating detection boxes on wrong location as well. This issue can be addressed with other methods like ensemble [27] or attention [48] module. The ensemble model [27] method allows using two models with complementary information, which may be useful when detecting overlapping pigs by training the second model on overlapping pigs and combining their features. Attention [48] may also help as it can focus its detection process on region with overlapping features on it.

Another problem to be studied is the accuracy difference between tinyYOLOv4 [41] and tinyYOLOv7 [42]. According to prior work, tinyYOLOv7 shows higher detection accuracy on COCO dataset that has 80 classes compared to tinyYOLOv4, which shows opposite data on Table 8. Additional research will be done to analyze the reason behind lower accuracy on the more recent model with pig dataset that has one class.

## 6. Conclusions

Accurate object detection is crucial when obtaining useful information by monitoring pigs on actual farms. However, despite recent advances in the accuracy of object detectors for single images, there are still problems (i.e., False Negative errors, and False Positive errors) occurring due to overlapping phenomena such as invisible parts of the pigs covered by facilities.

In this study, we proposed a method to improve the accuracy of deep learning-based pig detectors by utilizing the characteristic of video data (i.e., a sequence of images) and image processing techniques obtained from static cameras installed in pig pens. In other words, the cyclic structure method can continuously improve the accuracy of object detection by employing received results for image processing and applying these image processing results to correct the output of object detection. Consequently, a background image can be improved by continuously receiving box unit object detection results and updating the background extraction parameters, thus locating occluding objects such as feeding facilities. Finally, it is possible to generate composite images that has information of each background and facility location within an image and it can reduce the FN and FP errors for the single image by verifying and correcting the object detection result using the facility information. Addressing the problem of accuracy reduction problem on facility occlusion cases shows novelty as previous research on pig object detection has not done before.

Our proposed method improved accuracy from 84% to 94% compared to the baseline tinyYOLOv4. It also exhibited an improvement in accuracy from 76% to 90% for an external dataset, compared with the baseline tinyYOLOv4. In both cases, accuracy was improved without compromising processing speed, thus maintaining the real-time requirements. This shows practical significance as it can also be used in any farm having any sort of occluding facility with static cameras with high detection accuracy that meets the real-time requirement. In a future study, we intend to conduct an experiment on additional accuracy improvement methods, such as ensemble models [27] or directly inputting the image processing results into the detector in the form of an attention map. Furthermore, although our study alleviated the decrease in accuracy owing to occlusion, it did not address the case wherein pigs occlude each other. Therefore, our subsequent research will aim to address this issue.

## Figures and Tables

**Table 1:** Some of the recent results for group-housed pig detection (published during 2013–2022).

Background and Facility Information Utilization	Year	Detection Technique	Accuracy Reported	Reference
No	2013	Image Processing	88.70% ^※^	[3]
		Image Processing	93.30% ^※^	[4]
		Image Processing	89.80% ^※^	[5]
	2014	Image Processing	-	[6]
		Image Processing	99.00% ^※^	[7]
		Image Processing	89.90% ^※^	[8]
	2015	Image Processing	95.48% ^※^	[9]
		Image Processing	-	[10]
	2016	Image Processing	88.60%~94.50% ^※^	[11]
		Image Processing	90.20%~95.70% ^※^	[12]
		Image Processing	-	[13]
	2017	Image Processing	-	[14]
		Image Processing	94.47% ^※^	[15]
	2018	Deep Learning	89.58% ^※^	[16]
		Deep Learning	-	[17]
	2019	Deep Learning	-	[18]
		Image Processing + Deep Learning	86.80% ^※^	[19]
		Image Processing + Deep Learning	92.00%~95.00% ^※^	[20]
		Image Processing + Deep Learning	77.10%~98.10% ^※^	[21]
	2020	Deep Learning	94.70% ^※^	[22]
		Deep Learning	96.50%~97.60% ^※^	[23]
		Deep Learning	98.00%~99.80% ^※^	[24]
		Deep Learning	67.70%~87.40% ^※^	[25]
		Image Processing + Deep Learning	95.00%^※^	[26]
	2021	Deep Learning	94.33%^※^	[27]
		Deep Learning	-	[28]
		Deep Learning	58.00%~84.00% ^※^	[29]
		Deep Learning	93.1%^※^	[30]
		Image Processing + Deep Learning	-	[31]
		Image Processing + Deep Learning	92.45% ^※^	[32]
		Image Processing + Deep Learning	-	[33]
	2022	Deep Learning	99.44% ^※^	[34]
		Deep Learning	-	[35]
		Image Processing + Deep Learning	82.80%~99.50% ^※^	[36]
Yes	2022	Image Processing + Deep Learning	94.45% *	Proposed

^※^ The accuracy may vary due to the test dataset which the detection accuracy is based on. * The accuracy is for a difficult case that contains more than 20 pigs occluded behind facility within a pig pen.

**Table 2:** Detection accuracy results for Hadong dataset with tinyYOLOv4 [41].

Type	Train Dataset (Hadong)	Test Dataset (Hadong)	TP	FP	FN	Precision	Recall	Accuracy (AP_0.5_)
Baseline	Color Image	Color Image	3729	517	864	0.88	0.81	84.39
Proposed StaticPigDet	Composite Image A + B + C + D	Composite Image A	3885	385	385	0.91	0.91	94.02
		Composite Image B	3916	398	354	0.91	0.92	94.19
		Composite Image C	3882	272	388	0.93	0.91	94.44
		Composite Image D	3868	332	402	0.92	0.91	94.45

**Table 3:** Detection accuracy results for Hadong dataset with tinyYOLOv7 [42].

Type	Train Dataset (Hadong)	Test Dataset (Hadong)	TP	FP	FN	Precision	Recall	Accuracy (AP_0.5_)
Baseline	Color Image	Color Image	3967	2135	626	0.65	0.86	86.70
Proposed StaticPigDet	Composite Image A + B + C + D	Composite Image A	3709	500	561	0.88	0.87	91.83
		Composite Image B	3725	491	545	0.88	0.87	92.24
		Composite Image C	3672	373	598	0.91	0.86	91.78
		Composite Image D	3585	392	685	0.90	0.84	91.04

**Table 4:** Detection accuracy results for individual composite image dataset A~D with tinyYOLOv4 [41].

Train Dataset (Hadong)	Test Dataset (Hadong)	TP	FP	FN	Precision	Recall	Accuracy (AP_0.5_)
Composite Image A	Composite Image A	3632	479	638	0.88	0.85	89.88
Composite Image B	Composite Image B	3657	819	613	0.82	0.86	86.77
Composite Image C	Composite Image C	3686	810	584	0.82	0.86	87.78
Composite Image D	Composite Image D	3463	656	807	0.84	0.81	83.77

**Table 5:** Detection accuracy results for individual composite image dataset A~D with tinyYOLOv7 [42].

Train Dataset (Hadong)	Test Dataset (Hadong)	TP	FP	FN	Precision	Recall	Accuracy (AP_0.5_)
Composite Image A	Composite Image A	3620	1034	650	0.78	0.85	86.46
Composite Image B	Composite Image B	3703	1544	567	0.71	0.87	87.45
Composite Image C	Composite Image C	3668	812	602	0.82	0.86	88.30
Composite Image D	Composite Image D	3621	815	649	0.82	0.85	86.60

**Table 6:** Detection accuracy results for Chungbuk dataset with tinyYOLOv4 [41].

Type	Train Dataset (Hadong)	Test Dataset (Chungbuk)	TP	FP	FN	Precision	Recall	Accuracy (AP_0.5_)
Baseline	Color Image	Color Image	764	42	958	0.95	0.44	75.86
Proposed StaticPigDet	Composite Image A + B + C + D	Composite Image A	1543	1276	179	0.55	0.90	81.30
		Composite Image B	1526	919	196	0.62	0.89	77.41
		Composite Image C	1509	235	213	0.87	0.88	88.71
		Composite Image D	1472	112	250	0.93	0.85	90.25

**Table 7:** Detection accuracy results for Chungbuk dataset with tinyYOLOv7 [42].

Type	Train Dataset (Hadong)	Test Dataset (Chungbuk)	TP	FP	FN	Precision	Recall	Accuracy (AP_0.5_)
Baseline	Color Image	Color Image	1270	2168	452	0.37	0.74	63.15
Proposed StaticPigDet	Composite Image A + B + C + D	Composite Image A	1280	1612	442	0.44	0.74	65.41
		Composite Image B	1282	1220	440	0.51	0.74	68.77
		Composite Image C	1248	979	474	0.56	0.72	65.71
		Composite Image D	1371	709	351	0.66	0.80	77.76

**Table 8:** Comparison of average performance for Hadong pig pen on a TX-2 [46].

Method		Accuracy (AP_0.5_)	Speed (FPS)	Integrated Performance = Accuracy × Speed
tinyYOLOv4 [41]	Baseline	84.4	36.3	3063.7
	Proposed	94.5	36.1	3411.4
tinyYOLOv7 [42]	Baseline	86.7	34.6	2999.8
	Proposed	92.2	34.2	3153.2

## Data Availability

Background/Facility/NPPS visualization: https://youtu.be/HX8yaHz86L8 (accessed on 23 October 2022).

## References

[1] OECD (2021). Meat Consumption (Indicator). https://www.oecd-ilibrary.org/agriculture-and-food/meat-consumption/indicator/english_fa290fd0-en.

[2] Jiangong L., Green-Miller A., Hu X., Lucic A., Mahesh M., Dilger R., Condotta I., Aldridge B., Hart J., Ahuja N. (2022). Barriers to computer vision applications in pig production facilities. Comput. Electron. Agric..

[3] Kashiha M., Bahr C., Ott S., Moons C., Niewold T., Ödberg F., Berckmans D. (2013). Automatic identification of marked pigs in a pen using image pattern recognition. Comput. Electron. Agric..

[4] Tu G., Karstoft H., Pedersen L., Jørgensen E. (2013). Foreground detection using loopy belief propagation. Biosyst. Eng..

[5] Kashiha M., Bahr C., Ott S., Moons C. (2013). Automatic monitoring of pig activity using image analysis. Livest. Sci..

[6] Ott S., Moons C., Kashiha M., Bahr C., Tuyttens F., Berckmans D., Niewold T. (2014). Automated video analysis of pig activity at pen level highly correlates to human observations of behavioural activities. Livest. Sci..

[7] Chung Y., Kim H., Lee H., Park D., Jeon T., Chang H. (2014). A cost-effective pigsty monitoring system based on a video sensor. KSII Trans. Internet Inf..

[8] Kashiha M., Bahr C., Ott S., Moons C., Niewold T., Tuyttens F., Berckmans D. (2014). Automatic monitoring of pig locomotion using image analysis. Livest. Sci..

[9] Tu G., Karstoft H., Pedersen L., Jørgensen E. (2015). Illumination and reflectance estimation with its application in foreground. Sensors.

[10] Guo Y., Zhu W., Jiao P., Ma C., Yang J. (2015). Multi-object extraction from topview group-housed pig images based on adaptive partitioning and multilevel thresholding segmentation. Biosyst. Eng..

[11] Nasirahmadi A., Hensel O., Edwards S., Sturm B. (2016). Automation detection of mounting behaviours among pigs using image analysis. Comput. Electron. Agric..

[12] Lee J., Jin L., Park D., Chung Y. (2016). Automatic recognition of aggressive behavior in pigs using a kinect depth sensor. Sensors.

[13] Gronskyte R., Clemmensen L., Hviid M., Kulahci M. (2016). Monitoring pig movement at the slaughterhouse using optical flow and modified angular histogram. Biosyst. Eng..

[14] Buayai P., Kantanukul T., Leung C., Saikaew K. (2017). Boundary detection of pigs in pens based on adaptive thresholding using an integral image and adaptive partitioning. CMU J. Nat. Sci..

[15] Kim J., Chung Y., Choi Y., Sa J., Kim H., Chung Y., Park D., Kim H. (2017). Depth-based detection of standing-pigs in moving noise environments. Sensors.

[16] Zhang L., Gray H., Ye X., Collins L., Allinson N. (2018). Automatic individual pig detection and tracking in surveillance videos. arXiv.

[17] Brünger J., Traulsen I., Koch R. (2018). Model-based detection of pigs in images under sub-optimal conditions. Comput. Electron. Agric..

[18] Tian M., Guo H., Chen H., Wang Q., Long C., Ma Y. (2019). Automated pig counting using deep learning. Comput. Electron. Agric..

[19] Li B., Liu L., Shen M., Sun Y., Lu M. (2019). Group-housed pig detection in video surveillance of overhead views using multi-feature template matching. Biosyst. Eng..

[20] Nasirahmadi A., Sturm B., Edwards S., Jeppsson K., Olsson A., Müller S., Hensel O. (2019). Deep learning and machine vision approaches for posture detection of individual pigs. Sensors.

[21] Psota E., Mittek M., Pérez L., Schmidt T., Mote B. (2019). Multi-Pig Part Detection and Association with a Fully-Convolutional Network. Sensors.

[22] Hong M., Ahn H., Atif O., Lee J., Park D., Chung Y. (2020). Field-applicable pig anomaly detection system using vocalization for embedded board implementations. Appl. Sci..

[23] Chen C., Zhu W., Oczak M., Maschat K., Baumgartner J., Larsen M., Norton T. (2020). A computer vision approach for recognition of the engagement of pigs with different enrichment objects. Comput. Electron. Agric..

[24] Alameer A., Kyriazakis I., Bacardit J. (2020). Automated recognition of postures and drinking behaviour for the detection of compromised health in pigs. Sci. Rep..

[25] Riekert M., Klein A., Adrion F., Hoffmann C., Gallmann E. (2020). Automatically detecting pig position and posture by 2D camera imaging and deep learning. Comput. Electron. Agric..

[26] Brünger J., Gentz M., Traulsen I., Koch R. (2020). Panoptic segmentation of individual pigs for posture recognition. Sensors.

[27] Ahn H., Son S., Kim H., Lee S., Chung Y., Park D. (2021). EnsemblePigDet: Ensemble deep learning for accurate pig detection. Appl. Sci..

[28] Huang E., Mao A., Gan H., Ceballos M., Parsons T., Xue Y., Liu K. (2021). Center clustering network improves piglet counting under occlusion. Comput. Electron. Agric..

[29] Riekert M., Opderbeck S., Wild A., Gallmann E. (2021). Model selection for 24/7 pig position and posture detection by 2D camera imaging and deep learning. Comput. Electron. Agric..

[30] Hu Z., Yang H., Lou T. (2021). Dual attention-guided feature pyramid network for instance segmentation of group pigs. Comput. Electron. Agric..

[31] Hegde S., Gangisetty S. (2021). Pig-net: Inception based deep learning architecture for 3d point cloud segmentation. Comput. Graphics..

[32] Shao H., Pu J., Mu J. (2021). Pig-posture recognition based on computer vision: Dataset and exploration. Animals.

[33] Ocepek M., Žnidar A., Lavrič M., Škorjanc D. (2022). DigiPig: First developments of an automated monitoring system for body, head, and tail detection in intensive pig farming. Agriculture.

[34] Kim J., Suh Y., Lee J., Chae H., Ahn H., Chung Y., Park D. (2022). EmbeddedPigCount: Pig counting with video object detection and tracking on an embedded board. Sensors.

[35] Bo Z., Atif O., Lee J., Park D., Chung Y. (2022). GAN-Based video denoising with attention mechanism for field-applicable pig detection system. Sensors.

[36] Ji H., Yu J., Lao F., Zhuang Y., Wen Y., Teng G. (2022). Automatic position detection and posture recognition of grouped pigs based on deep learning. Agriculture.

[37] Zhao Z., Zheng P., Xu S., Wu X. (2018). Object detection with deep learning: A review. IEEE Access.

[38] Redmon J., Divvala S., Girshick R., Farhadi A. You only look once: Unified, real-time object detection. Proceedings of the IEEE Conference on Computer Vision and Pattern Recognition.

[39] Redmon J., Farhadi A. YOLO9000: Better, faster, stronger. Proceedings of the IEEE Conference on Computer Vision and Pattern Recognition.

[40] Redmon J., Farhadi A. (2018). YOLOv3: An incremental improvement. arXiv.

[41] Bochkovskiy A., Wang C., Liao H. (2020). Yolov4: Optimal speed and accuracy of object detection. arXiv.

[42] Wang C., Bochkovskiy A., Liao H. (2022). YOLOv7: Trainable bag-of-freebies sets new state-of-the-art for real-time object detectors. arXiv.

[43] Open Source Computer Vision: ‘OpenCV’. http://opencv.org.

[44] Zuiderveld K. (1994). Contrast Limited Adaptive Histogram Equalization.

[45] Hanwha Surveillance Camera. https://www.hanwhasecurity.com/product/qno-6012r/.

[46] NVIDIA NVIDIA Jetson TX2. http://www.nvidia.com/object/embedded-systems-dev-kitsmodules.html.

[47] Intel Intel RealSense D435. https://www.intelrealsense.com/depth-camera-d435.

[48] Vaswani A., Shazeer N., Parmar N., Uszkareit J., Jones L., Gomez A., Kaiser G., Polosukhin I. Attention is all you need. Proceedings of the NeurIPS.

